# Addendum to Failed Examinees' Legal Challenge over the Clinical Skill Test in the Korean Medical Licensing Examination

**DOI:** 10.3352/jeehp.2010.7.6

**Published:** 2010-12-14

**Authors:** Sun Huh

**Affiliations:** Department of Parasitology, College of Medicine and Institute of Medical Education, Hallym University, Chuncheon, Korea.

In December 8, 2010, the trial concerning the legal challenge over the clinical skill test in the Korean Medical Licensing Examination that was administered beginning in February 2010 ended [1]. The judge of the Administrative Court in Seoul reached a verdict, rejecting the suit brought by the examinees who failed. The summary of the court decision [2] is translated by the present author as follows: *The items of the clinical performance test were designed to evaluate the clinical practice capacity. To evaluate the examinees' capacity, a physician's direct oversight is not essential. Well-trained standardized patients can do it. Other developed countries accept standardized patients as evaluators of clinical performance. In addition, standardized patients participate in the grading of the examinee's performance. It cannot be concluded that only the physician can grade examinees consistently. The activities of the National Health Personnel Licensing Examination Board cannot be said to be against the discretionary power of examination by weakening the rationality or interests of justice*.

The above decision was as expected. Activities of professional organizations or societies are usually not overturned in court if they do not violate common sense. However, it is only a matter of time before the licensing test is challenged in court again, and dealing with such trivial lawsuits is a waste of time and human resources. We should design and construct a system to prevent lawsuits that distract the leaders of our profession in this country from focusing on improving the quality of care for our patients through fair and rigorous medical licensing.
